# 

**Published:** 1893-06-17

**Authors:** 


**OUR NEW OFFICES**
428, Strand, W.C., will henceforth be the Office of this Journal.

**Notices of Births, Deaths, and Marriages,** are inserted in this column at a charge of 2s. 6d. for an announcement not exceeding four lines.

### NOTICE TO CORRESPONDENTS.

All MS., Letters, Books for Review, and other matters intended for the Editor should be addressed THE EDITOR, THE LODGE, PORCHESTER SQUARE, LONDON, W.

The Editor cannot undertake to return rejected M.S., even when accompanied by stamped directed envelope.

**Notice to Advertisers and Subscribers.**

All Advertisements, Orders for Copies of THE HOSPITAL, and Business Communications should be addressed to The Publisher, *not to the Editor*, THE HOSPITAL, 428, Strand, W.C.

The Cost of Subscription, post free, is as follows:—Per week, 2½d.; per quarter, 2s. 6d.; per half-year, 5s.; per annum, 8s. 6d. Foreign:—Per annum, 10s. 8d.; payable, in all cases, in advance.

**"Burdett's Hospital Annual," 1893.**

Fourth year of publication. 700 pp. Boards, gilt lettering. A most complete guide to British, Colonial, Indian, and American Hospitals, Dispensaries, Nursing and Convalescent Institutions, and Asylums. Price 5s. Published by THE SCIENTIFIC PRESS (Limited), 428, Strand, W.C.

**NOTICE.—The Index for Vol. XIII. (ending March 1893) is on sale at the Office of this Journal, price 2½d., post free.**

### SCRAPS AND GLEANINGS.

Fourteen cases of deaths from drowning were registered recently in the weekly report of the metropolis.

It is stated that there are 3,000 more paupers in London at the present season than at the same time last year.

Sir Spencer Wells has lately been visiting Glasgow, and lecturing on "The Prevention of Preventable Disease."

The report of the Society for the Relief of Widows and Orphans of Medical Men states that the expenditure had been £3,265, and a deficit exists of £73.

The Salvation Army shelters are being held responsible in certain minds for a very possible spread of infectious disease through these favourite resorts.

The King of the Belgians appeared in person at Brussels lately to lay the foundation stone of the new buildings which are to serve as a Workman's Home Society.

The Library Commissioners of St. Saviour's have accepted the tender of a firm of builders for the erection of a new public library for Southwark at a cost of £4,467.

The examination premises of the Society of Apothecaries in London are being enlarged to meet the requirements of the numerous candidates who present themselves.

Dr. Haffkine and Mr. Hankin are now continuing their anti-cholera vaccination at Lucknow every fifth day. In the first month of these operations 1,00[illegible] experiments were effected.

A Society of Magnetizers has lately appeared in Paris. Those patients who put themselves under the professional care of any member of the society will be treated without the aid of drugs.

At the Cochin Hospital, Paris, on May 30th, the two new wards, bearing the names of Lister and Pasteur, were opened. The ceremony was attended by Monsieur Pasteur, who found many words of praise for all the arrangements.

The Pilton Field site, which was selected for the Infectious Diseases Hospital at Leith, has not proved itself all that is desirable financially. The estimate for the establishment has mounted up by degrees, until now it stands at £35,[illegible]00.

At an inquest lately held at Bishop Auckland, the Coroner remarked that this was one more case where life might have been saved had there been local facilities for the proper treatment in case of illness. There is no hospital in the vicinity.

The debt on the newly-built wards of the Hospital for Sick Children, Great Ormond Street, now amounts to £12,000. Their Royal Highnesses the Prince and Princess of Wales have promised to receive purses on the occasion of the opening ceremony.

Energetic steps at length are being taken to provide hospital accommodation for Blackpool. It is said that the foundation stone will be laid in a few weeks, and the date anticipated for the completion of the buildings is in early November.

A public inquiry was opened on May 30th at the Board-room of the Lewisham Workhouse respecting the proposal to purchase Hither Green Lodge and Wilderness House estates at Lewisham for the purpose of erecting the Fever Hospital thereon.

It has been urgently pressed upon the Sowerby Local Board to obtain more suitable premises for the Small-pox Hospital before it is too late. The present site which has been selected has no water in the vicinity, either for drinking or sanitary purposes; also it is situated on the high road and close to inhabited dwellings.

The report of the Adelaide Hospital, Dublin, at the annual meeting states that the pressure on the accommodation was so great that the Committee were only with the greatest difficulty enabled to give the wards the necessary cleaning, and in the Victoria House, devoted to fever cases, the cleaning had to be postponed until after the year had expired.

### Medical Vacancies and Appointments.

### APPOINTMENTS.

Annie M. S. Anderson, M.B.Lond., appointed Resident Medical Officer to the New Hospital for Women, Euston Road, N.W. Mr. W. H. Apthorpe, appointed Medical Officer and Public Vaccinator for the No. 5 District of the Cuckfield Union. W. Rushton Ashworth, L.R.C.P.Lond., M.R.C.S., appointed Resident Surgeon to the Costa Rica Railway Company's Hospital at Port Limon. J. Atkinson, M.B., C.M.Edin., appointed Senior Resident Medical Officer to the North-West London Hospital, Kentish Town Road. G. A. H. Barton, M.R.C.S.Eng., appointed Medical Officer for the Moreland Bishop District of the Crediton Union. J. Cagney, M.A., M.D., M.R.C.P., appointed an Assistant Physician at the North-West London Hospital. T. M. Cann, M.R.C.S.Eng., appointed Medical Officer for the Rodmell, Ilford, and Kingston District of the Newhaven Union. Dr. Cressy, appointed Medical Officer for the Wroughton District of the Highworth and Swindon Union. J. Cromie, L.R.C.P., L.R.C.S. Edin., reappointed Medical Officer to the Blyth Port Sanitary Authority. H. B. Donkin, M.A., M.B.Oxon., F.R.C.P.Lond., appointed Joint Lecturer on Medicine at the Westminster Hospital Medical School. Eleanor L. Fleury, M.B., B.Ch.Irel., appointed Clinical Assistant to the Richmond Asylum, Dublin. W. F. Foster, M.R.C.S.Eng., appointed Medical Officer of Health for the Newport Urban Sanitary District of the Isle of Wight Union. E. Gaylor, L.R.C.P., L.M.Edin., L.F.P.S.Glasg., reappointed Medical Officer of Health for the Ripley Urban Sanitary District. A. Harris, M.B.Edin., appointed Medical Officer of Health for the Thetford Union. E. W. Hope, M.D., appointed *pro tem.* Medical Officer of Health to the Liverpool Port Sanitary Authority. J. Hutchinson, jun., F.R.C.S.Eng., appointed Surgeon to the Out-Patients at the London Hospital. J. A. Johns, M.B., B.Ch., appointed Resident Medical Officer to the Westmoreland Lock Hospital, Dublin. T. H. Kellock, M.A., M.B., B.C., F.R.C.S., L.R.C.P., appointed Resident Medical Superintendent of the Hospital for Sick Children, Great Ormond Street. N. F. Kendall, M.R.C.S., L.R.C.P.Lond., appointed Junior Resident Medical Officer to the North-West London Hospital, Kentish Town Road. A. E. Kennedy, L.R.C.P., L.M.Edin., L.F.P.S. Glasg., appointed Medical Officer for the Small-Pox Hospital at West Ham. J. Kerr, M.A., M.D., D.P.H.Cantab, appointed Honorary Assistant Medical Officer to the Bradford Infirmary. Dr. N. Manning appointed Medical Officer for the Combmartin Friendly Society. Dr. Murray appointed Consulting Surgeon to the Inverness District Asylum. W. Oliver, M.B., C.M.Edin., appointed a District Medical Officer for Waterhead, in the Township of Saddleworth. J. C. O'Raffety, L.R.C.P., L.R.C.S.Edin., appointed Medical Officer for the Waltham District of the Melton Mowbray Union. J. A. Ormerod, M.D.Oxon., F.R.C.P. Lond., M.R.C.S., appointed House-Physician to St. Bartholomew's Hospital. H. H. Phillips, M.R.C.S.Eng., L.R.C.P.Lond., appointed House-Physician to Charing Cross Hospital. J. J. Powell, M.R.C.S.Eng., L.S.A.Lond., L.R.C.P.Lond., appointed Medical Officer to St. Nicholas's Home for Crippled Children at Byfleet, near Weybridge. W. B. Pritchard, M.R.C.S., L.R.C.P. Lond., appointed Assistant Medical Officer to the Royal Infirmary, Manchester. Dr. J. Rowett appointed Medical Officer for the Highworth District of the Highworth and Swindon Union. J. Tatham, M.D.St.And., F.R.C.P.Lond., appointed Consulting Physician to the Hospital for Consumption, Brompton. Dr. Trotter appointed Medical Officer for the Brandon District of the Thetford Union. J. H. Whitehead, M.R.C.S.Eng., L.R.C.P.Lond., appointed House-Surgeon to Charing Cross Hospital.

### VACANCIES.

The following vacancies are announced this week, the amount of salary in each case being given after the name of the institution:

**Resident Medical Officers.**

Belgrave Hospital for Children, 79, Gloucester Street, S.W. House-Surgeon. Board, lodging, fuel, and lights provided. Applications, endorsed outside "House-Surgeon," to the Honorary Secretary, by June 21st.

**MEDICAL VACANCIES AND APPOINTMENTS—(continued)**

Bradford Infirmary and Dispensary. House-Surgeon; unmarried. Salary £10 per annum, with board and residence. Applications, endorsed "House-Surgeon," to William Maw, Secretary, by June 20th.

Burton-on-Trent Infirmary. House-Surgeon. Salary £130 per annum, with rooms, coals, and gas. Applications to the Honorary Secretary, James C. Grinling, by June 23rd.

City of London Hospital for Diseases of the Chest, Victoria Park, E. Assistant Physician; Fellow or Member of the Royal College of Physicians of London. Applications, with testimonials, by July 5th, 1893.

East London Hospital for Children, Glamis Road, Shadwell, E. House-Surgeon. Board and lodging provided. Applications to the Secretary by July 6th.

General Hospital, Birmingham. Assistant House-Surgeon. No salary; residence, board, and washing. Applications, with registration certificate, to the House Governor by July 1st.

Liverpool Dispensaries. Assistant Surgeon; unmarried. Salary £80 per annum, with apartments, board, and attendance. Applications to R. R. Gree e, Secretary, by July 24th.

Manchester Southern and Maternity Hospital. Resident House-Surgeon. Honorarium at the rate of £75 per annum. Applications to George William Fox, 53, Princess Street, Manchester, by June 16th.

Royal Alexandra Hospital for Sick Children, 10, Dyke Road, Brighton. House-Surgeon; doubly qualified. Salary £80 per annum, with board, lodging, and washing. Applications to the Chairman of the Medical Committee by June 17th.

Royal Free Hospital, Gray's Inn Road, W.C. Junior Resident Medical Officer. House-Surgeon; doubly qualified. Appointment for six months. Board, residence, and washing provided. Applications to the Secretary by June 19th.

Royal Southern Hospital, Liverpool. Junior House-Surgeon; doubly qualified. Salary 60 guineas a year, with board and residence. Applications to the Chairman of the Medical Board by June 17th.

Royal Victoria Hospital, Bournemouth. House Surgeon-Secretary Salary £100 per annum, and board. Applications to the Chairman of Committee by July 1st.

West London Hospital, Hammersmith Road, W. House-Physician and House-Surgeon. Board and lodging provided. Applications to R. J. Gilbert, Secretary-Superintendent, by June 21st.

**Non-Resident Medical Officers.**

Hospital for Consumption and Diseases of the Chest, Brompton. Dental Surgeon. Applications to the Secretary by June 21st.

Infirmary for Consumption and Diseases of the Chest and Throat, 26, Margaret Street, Cavendish Square, W. Physician in Ordinary. Applications to the Secretary by June 21st.

Royal College of Surgeons of England. Examiner in Dental Surgery. Applications to the Secretary by June 23rd.

Royal Victoria Hospital, Bournemouth. Ophthalmic Surgeon. Applications to the Secretary by July 1st.

University College, London. Professor of Midwifery and Obstetrics. Applications to the Secretary, J. M. Horsburgh, M.A., by June 20th.

Victoria Hospital for Sick Children, Queen's Road, Chelsea, S.W. Surgeon on the in-patient staff and Surgeon on the out-patient staff. Appointment for five years. Applications to Commander Blount, R.N., Secretary, by June 17th.

University of London, Burlington Gardens, W. Examiner in Surgery. Salary £200 per annum. Applications to the Registrar by June 24th.

West London Hospital, Hammersmith Road, W. Surgeon. In view of one of the Assistant Surgeons being elected, applications are invited for Assistant Surgeon. Applications to R. J. Gilbert, Secretary-Superintendent, by June 21st.

Westminster Hospital, Broad Sanctuary, S.W. Assistant Physician. Applications to House Committee before June 20th.

### MEDICAL APPOINTMENTS.

**ROYAL VICTORIA HOSPITAL, Bournemouth.**

**ROYAL FREE HOSPITAL, Gray's Inn Road.**

**BRADFORD INFIRMARY AND DISPENSARY.**

